# Dengue Reborn: Widespread Resurgence of a Resilient Vector

**DOI:** 10.1289/ehp.116-a382

**Published:** 2008-09

**Authors:** Melissa Lee Phillips

Dengue—a viral disease that can refer to both dengue fever and the more severe dengue hemorrhagic fever (DHF)—swept away records again this past spring as it raged across Brazil, infecting more than 160,000 people and killing more than 100. The reports were similar to those out of Southeast Asia in the summer of 2007, South America the previous spring, and India the fall before that. Although it may not be the most devastating of the mosquito-borne diseases—malaria strikes 10 times more people and yellow fever kills more of its victims—dengue has become a major public health concern for two reasons: the speed with which it is spreading and the escalating seriousness of its complications.

In the nineteenth century, dengue fever was a mild illness found in the tropics. Deaths were rare, and years passed between major epidemics. But since the mid-twentieth century, the range of the dengue virus has steadily broadened. In the last 50 years, its worldwide incidence has increased 30-fold, and various estimates posit that anywhere from one-third to nearly one-half of the world’s population are now at risk of becoming infected.

Moreover, today’s dengue infection is not what it once was. DHF, a complication of dengue infection that was not recognized until the 1950s (although cases probably occurred as early as 1870 in India), now appears in many dengue epidemics. In addition to the fever, rash, headache, and muscle and joint pain of classic dengue fever (which earned dengue its nickname of “breakbone fever”), DHF sometimes causes hemorrhaging that can lead to shock and even death. Epidemic DHF is now a leading cause of hospitalization and death among children in several Southeast Asian countries. Worldwide, of the 50 million dengue infections estimated by the World Health Organization (WHO) each year, there are 500,000 cases of DHF and 22,000 deaths, mainly among children.

Once considered mainly an Asian disease, dengue fever and DHF now also permeate the tropical Americas. Between 1995 and 2001, the number of dengue cases in the Americas doubled, according to the WHO. By 2007, the annual incidence there reached nearly 900,000 cases, with more than 25,000 people suffering DHF.

The dengue virus comes in four distinct serotypes. Individuals who become infected with one serotype obtain lifelong immunity against that serotype but not against the other three—and there is good evidence that a previous dengue infection increases the odds of developing DHF upon infection with a different serotype. “Somehow, having that prior infection enhances invasion of target cells by new dengue [serotypes],” explains Laura Harrington, a medical entomologist at Cornell University.

Dengue experts agree on many of the causes of the disease’s spread, including demographic changes and interruptions in vector control efforts. But some controversy has surfaced over whether climate change—often cited as a factor in broadening disease vector habitats—has had or will have anything to do with the virus’s expansion. “It’s too early to predict what effects global warming will have, if any,” says David Morens, senior scientific advisor at the National Institute of Allergy and Infectious Diseases (NIAID) in Bethesda, Maryland. “But it’s certainly something to be concerned about.”

## Re-emergence of a Disease

Several factors have assisted in the spread of dengue around the world. *Aedes aegypti*, the mosquito that is the chief carrier of the dengue virus, originated in Africa but migrated to other continents via the slave trade in the 1500s and 1600s, says Duane Gubler, director of the Asia-Pacific Institute of Tropical Medicine and Infectious Diseases at the University of Hawaii at Manoa in Honolulu. “As urban port cities developed, [the *Ae. aegypti*] mosquito became established and became highly adapted to humans,” he says. Accordingly, as the tropical developing world has become increasingly urbanized over the past few decades, *Ae. aegypti* have proliferated.

Whereas *Ae. aegypti* originally bred in small natural water bodies such as tree holes or rock pools, it now breeds successfully in water that accumulates in discarded trash such as bottles, plastic and cellophane packaging, and tires, as well as in domestic water storage containers that are common in places where people do not have easy access to a regular supply of clean water. *Ae. aegypti* also prefers to live inside buildings rather than outside. Finally, this mosquito prefers to feed on humans, meaning viral transmission is not diluted by the mosquito feeding on other animals as well. *Ae. aegypti* therefore is “perfectly adapted to the urban environment,” says Gubler.

During World War II, Japanese and Allied military movements spread viruses throughout Southeast Asia. In the aftermath of the war, “for the first time, several serotypes were coming together,” Harrington says, as people began to travel across the world more frequently. Subsequent economic boom and rapid urbanization in Southeast Asia led to conditions ideal for epidemics—cramped living quarters, low-quality housing, and poor management of water, sewage, and waste systems. Dengue’s progression from tropical nuisance to life-threatening epidemic reached a tipping point in the 1950s, when DHF was first reported in the Philippines and Thailand.

Meanwhile, on the other side of the globe, dengue had been largely eliminated in the Americas, mainly thanks to attempts to control urban yellow fever in the 1950s and 1960s. The Pan American Health Organization (PAHO), an international public health agency, initiated a campaign to rid Central and South American, Caribbean, and southern U.S. regions of *Ae. aegypti*, which also transmits yellow fever virus. By going after *Ae. aegypti* aggressively with the insecticide DDT and systematically eliminating its breeding areas, the campaign largely eradicated the vector from Central and South America, although not the Caribbean and southern United States, says Gubler. In the course of eradicating yellow fever, the efforts also squashed dengue transmission in the region.

DDT was banned in the United States in 1972. Coincidentally, says Gubler, *Ae. aegypti* eradication efforts were deemed successful and therefore largely abandoned, with resources redirected to other pressing issues of the day such as President Richard Nixon’s “War on Cancer.” Since then, *Ae. aegypti* has returned to nearly every region from which it was eliminated. “We have allowed *Aedes aegypti* to reinfest most if not all of the urban areas of tropical America,” says Gubler.

In 1981 a serotype of dengue imported from southeast Asia caused an outbreak of DHF in Cuba—the first DHF epidemic in the Americas. Since then, all four serotypes have spread throughout the Americas, causing DHF outbreaks and becoming endemic in many countries.

Increased global movement of people and cargo via air travel have undoubtedly assisted dengue’s growth, says Harrington. It is this movement that now allows multiple serotypes of dengue to encounter each other frequently, leading to the complications of DHF. And in the Americas, the reinvasion of *Ae. aegypti* after the lapse of eradication campaigns also contributed to dengue’s resurgence, Harrington says. “For those of us who work in dengue research, I think there’s a fairly strong consensus about what the major factors are [in dengue’s spread],” she says.

## The Climate Change Question

One factor, however, remains debatable: the effect of climate change on the dissemination of dengue. Like many vector-borne diseases, dengue fever shows a clear weather-related pattern: rainfall and temperatures affect both the spread of mosquito vectors and the likelihood that they will transmit virus from one human to another. In a cool climate, the virus takes so long to replicate inside the mosquito that most likely the mosquito would die before it actually has a chance to transmit the virus to another person, says senior research fellow Simon Hales of the University of Otago, New Zealand. “There’s a consensus that climate is one of the necessary factors that has to be right for dengue to be able to be transmitted,” Hales says. “Whether or not climate change will affect the spread of dengue is probably more contentious.”

Several studies have predicted that global climate change could increase the likelihood of dengue epidemics. In the 14 September 2002 issue of *The Lancet*, Hales and his colleagues published an empirical model of worldwide dengue distribution in which they reported that annual average vapor pressure (a measure of humidity) was the single climate factor that best predicted dengue fever distribution. They also used their model to predict likely effects of humidity on dengue distribution. If humidity were to remain at 1990 levels into the next century, a projected 3.5 billion people would be at risk of dengue infection in 2085, but assuming humidity increases as projected by the Intergovernmental Panel on Climate Change, the authors estimate that in fact 5.2 billion could be at risk.

Other work has reported correlations between dengue and climate variables such as El Niño, temperature, rainfall, and cloud cover. In March 2008, the United Nations Intergovernmental Panel on Climate Change released its *Fourth Assessment Report on Climate Change Impacts: Impacts, Adaptation and Vulnerability*, concluding that climate change could increase the number of people at risk of dengue infection.

But some dengue researchers feel that a case for a connection between dengue incidence and climate change has yet to be made. Global warming might influence dengue transmission “to the extent that it influences how water is managed and handled,” says Harrington, but temperature increases are probably not important for the virus’s expansion. “If you really sit down and look at the science, . . . there are no real hard data to show that [climate change is] having an effect,” she says.

There’s no argument that global warming is occurring, says Gubler, but as for the suggestion that it has played any role in the expansion of dengue, “It’s all hype. A lot of public health officials and a lot of policy makers use global warming as a cop-out, an excuse for not controlling a disease that is very preventable.”

In a plenary session at the May 2008 annual meeting of the American Institute of Biological Sciences, Gubler urged that policy makers not focus on climate change but resume addressing the chief known drivers of dengue’s spread—namely, population growth, urbanization, and modern transportation. Importantly, he said, “we need political will. With political will, we may get the economic support that we need to do the research to develop effective prevention and control strategies.”

But even as there is no documentation that climate change is influencing the spread of dengue, Hales counters there also is no proof regarding many other factors claimed responsible for increased dengue—such as urbanization, population increase, and heightened travel—and that no published studies have attempted to assess the relative importance of these factors in comparison to temperature trends. It is not controversial, he adds, that dengue is highly temperature-sensitive, citing work published by Douglas M. Watts and colleagues in the January 1987 issue of *The American Journal of Tropical Medicine and Hygiene* showing that temperature-induced variations in how efficiently *Ae. aegypti* transmits the dengue virus may be “a significant determinant” in the annual cyclic pattern of DHF epidemics in Bangkok.

As for whether dengue is very preventable, Hales points to the example of Singapore, where dengue persists despite the best efforts of this wealthy country with its well-developed public health infrastructure and vector control. Hales concedes that it’s too soon to say for sure whether climate change is promoting the spread of dengue, but that “other things being equal, we would expect [the disease] to spread with projected climate change.” If Earth warms as expected, “then a larger area of the planet’s surface will be climatically suitable for dengue,” he says.

Global transport has helped another dengue vector spread to new territory. An article in the September 1987 issue of the *Journal of the American Mosquito Control Association* noted that the Asian tiger mosquito, *Aedes albopictus*, spread worldwide through the international trade in used tires. Over the past 25 years, the relatively cold-hardy *Ae. albopictus* has invaded many U.S. states, and rising average temperatures raise the possibility that the vector could move even further north.

*Ae. albopictus* is occasionally an important dengue vector in rural and suburban areas in Southeast Asia, says Philip McCall, a medical entomologist at the Liverpool School of Tropical Medicine, United Kingdom, and it was also behind Hawaii’s 2001 outbreak of 122 cases on the island of Maui. But Gubler says it is a mistake to assume that dengue epidemics will necessarily result from the spread of *Ae. albopictus*. Although this mosquito has been shown to be a highly efficient carrier in controlled experiments, it is far less so in real-world situations, he explains, mainly because it feeds on both humans and nonhuman animals, and it prefers rural environments to urban settings. If *Ae. albopictus* populations were to displace *Ae. aegypti*, then that could actually lead to reduced risk of dengue transmission, Gubler says.

## A Disease of Poverty

Dengue is a disease of poverty, Hales says. “In the places where it’s really rife, typically urban shantytowns, people have got very poor services,” he explains. “Waste is piling up in the street. There’s no running water, so people have to collect water in vessels, which then breed mosquitoes. The people have got terrible housing, so they’re not able to protect themselves from getting bitten. And they’re living in very close proximity. It’s the perfect recipe for a huge epidemic.”

Even if today’s temperate latitudes did become more suitable for dengue transmission, Gubler says, most of those regions are more developed and have good enough housing and water supply that dengue epidemics would remain unlikely. The standard of living in the United States will likely prevent any major dengue epidemics. “The United States is not going to have major epidemics of vector-borne diseases unless we allow our public health system to deteriorate completely,” Gubler says.

But professor Peter Hotez of The Sabin Vaccine Institute and George Washington University worries about the effect of dengue and other diseases he calls “neglected infections of poverty” on the poorest people in the United States. “There’s always been this reservoir of people at risk, and my concern is that, because they’re poor and voiceless, we ignore them,” he says.

Although dengue is endemic in Puerto Rico (where it has caused epidemics since the 1960s), it is absent from most of the continental United States, except in travelers returning from tropical locales. However, the disease appears occasionally along the U.S.–Mexico border, Hotez says. Along the border, reported incidence is much higher in the Mexican states than the U.S. ones, probably because of different living standards—window screens, air conditioning, and effective sanitation may help keep dengue at bay on the U.S. side, Hotez says.

However, a study published in the October 2007 issue of *Emerging Infectious Diseases* found that dengue incidence was surprisingly high in the border town of Brownsville, Texas. The researchers found evidence of past dengue infection in 40% of Brownsville residents. People with low income, no air conditioning, and poor street drainage were most likely to have suffered infection.

In a review published 25 June 2008 in *PLoS Neglected Tropical Diseases*, Hotez estimated as many as 200,000 U.S. cases of dengue fever occur each year, “but the estimates are pretty wide-ranging,” he says. There have so far been few reports of DHF in the United States. However, Hotez points out that DHF outbreaks have happened as close as Cuba; therefore, he says, “so there’s every reason to believe that it could happen in the United States.”

Many of the people at risk of dengue infection in the United States are members of minority groups, Hotez says—something that also applies to other infections that many people think of as “tropical” disease. Besides dengue, low-income Hispanic communities near the Mexican border are also at risk of Chagas disease, cutaneous leishmaniasis, and cysticercosis, an infection caused by ingesting tapeworm eggs, which is now a leading cause of epilepsy and seizures in areas around the U.S.–Mexico border. Many of these infections have been around for a while, Hotez says; however, “We’ve just ignored and neglected them because we tend not to pay attention to the plight of the poor and underrepresented minorities.”

Hotez suspects that people living in other areas prone to neglected infections—especially the post-Katrina Gulf Coast and elsewhere in the Mississippi River delta—are at some risk of dengue, but few data have been collected. “It’s not clear how many cases of dengue infection there are each year in the United States,” he says. “We’re not doing aggressive surveillance.”

## Curbing Dengue’s Expansion

Researchers are coming at dengue from a variety of angles to try to curb the virus’s spread. There are no available vaccines or antivirals for dengue infection, leaving mosquito control as the only current method for prevention and control.

“Ultimately, we need a vaccine for dengue,” says Harrington. “That’s probably the only way that we’re going to be able to have a significant impact.” Dengue vaccine development has proven challenging, largely because of the four different virus serotypes in circulation. Because DHF usually occurs when an individual already has immunity against one dengue serotype, researchers fear that vaccines that fail to provide equal immunity against all four serotypes may actually predispose people to hemorrhagic complications if they encounter a novel serotype after being vaccinated. “That has really slowed the development of the dengue vaccine,” Hotez says.

Currently, researchers at the Korea-based Pediatric Dengue Vaccine Initiative, chaired by Gubler and funded by the Bill & Melinda Gates Foundation, are facilitating the development of several different technologies to overcome those obstacles, for example, by helping some companies with clinical trials, establishing field sites, and working with developing countries to create the infrastructure to manage eventual vaccine distribution.

As part of the Grand Challenges in Global Health program, also funded in part by the Gates Foundation, researchers are creating mosquitoes that are genetically incapable of transmitting the dengue virus. About a dozen scientists worldwide are tackling different facets of the project, says Harrington, whose laboratory is assessing whether the transgenic strains are likely to outcompete *Ae. aegypti* for resources and mates in the wild. “If you could dream about something that could really make an impact, this would be it,” she says.

For now, dengue control still relies heavily on controlling the mosquito that transmits it. McCall and his colleagues have been running studies in Latin America and Southeast Asia to judge the effectiveness of household-based insecticide-treated materials (such as window curtains) and domestic water container covers as foils to the mosquito carriers and dengue transmission. Also, scientists from Vietnam and Australia reported in the January 2005 issue of *The American Journal of Tropical Medicine and Hygiene* that cultivating a natural predator of *Aedes* mosquitoes, the tiny crustacean *Mesocyclops*, in water storage containers virtually eliminated *Ae. aegypti* populations. “I was amazed,” McCall says. “I’m often skeptical about biological control, but in Vietnam, when used in combination with clean-up and education campaigns, this seems to have been spectacularly successful.”

Research continues on all fronts, adding to the collective knowledge about dengue transmission and, in some cases, challenging long-held assumptions. A mathematical model published by Suwich Thammapalo and colleagues in the 12 February 2008 issue of *Proceedings of the National Academy of Sciences* showed that decreasing dengue transmission may sometimes cause an increase in cases of DHF. The model’s predictions were boosted by epidemiologic data from Thailand that were later published 16 July 2008 in *PLoS Neglected Tropical Diseases*. The authors of both papers speculate that the effect may arise from a brief, transient cross-protection that people experience when infected with one serotype of dengue. At very high levels of dengue transmission, people could then have immunity to all four serotypes of the virus. If transmission is reduced moderately, this cross-immunity would also be reduced. The results are controversial, McCall says, “but many in the field believe it to be the case.”

Not everyone agrees, however, and Harrington summarizes some of the concerns about the paper. The authors based their conclusions on the relationship between dengue infection and transmission as a function of mosquito abundance as measured using the Breteau index, which reflects the number of water containers with mosquito larvae in 100 randomly selected houses in a community. But the Breteau index is a poor estimate of mosquito abundance, she says, and it rarely indicates what species is abundant. Moreover, it does not provide a large enough sample size to be powerful and meaningful.

“This type of work is a prime example of scientists working in isolation,” she says. “It highlights the need for cross-collaborative work on models for dengue ecology and epidemiology where biologically meaningful models can be developed.”

According to Hales, some of the most promising solutions may not directly involve mosquito eradication and may have little to do with technology. “What people [at risk] need is a decent environment in which to live,” he says. “If we had a dengue vaccine, most likely those people wouldn’t be able to afford it anyway. I’m not saying don’t look for a vaccine, but that’s probably not a short-term answer for the problem for these people.”

## Figures and Tables

**Figure:**
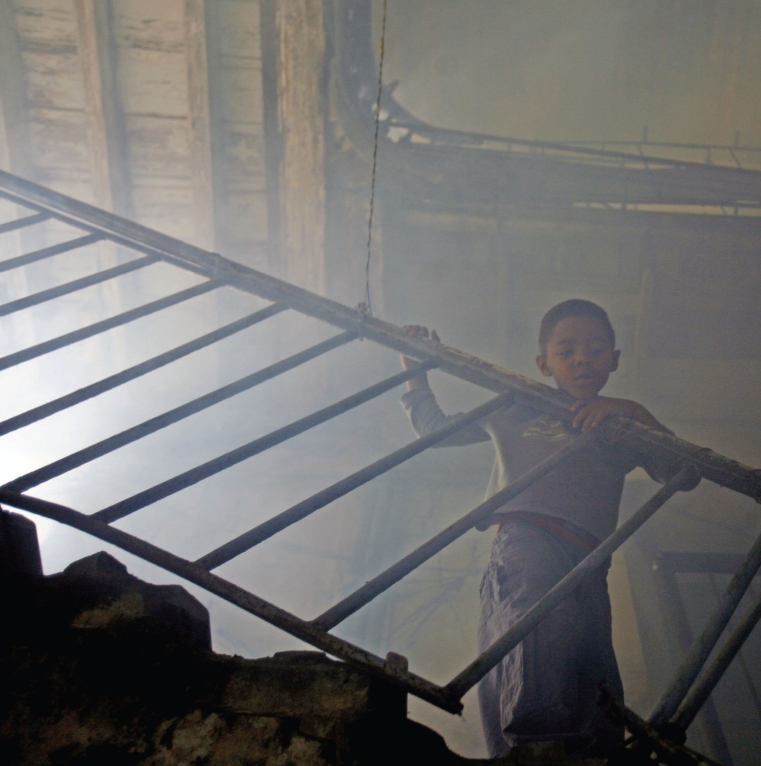
A child watches as a worker fumigates to prevent dengue fever and other mosquito-borne diseases, Old Havana, Cuba, January 2008.

**Figure:**
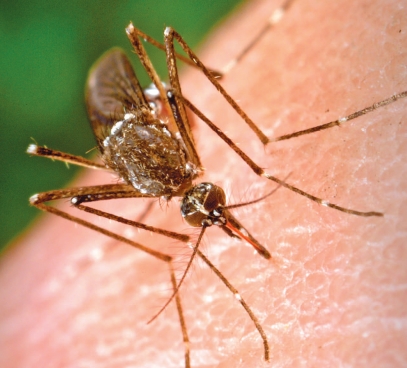
*Aedes aegypti*, the primary vector for dengue, has become perfectly adapted to the urban environment. In the wake of discontinued eradication efforts, *Ae. aegypti* has reinfested nearly every region from which it was eliminated.

**Figure:**
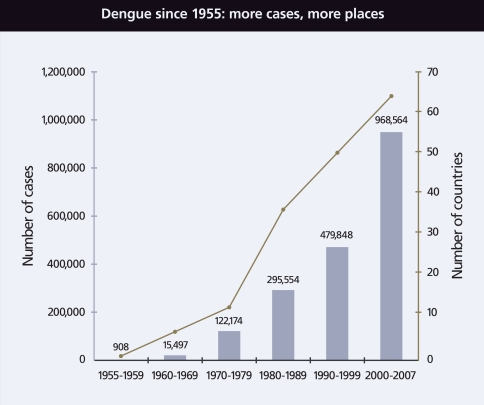
In the wake of rapid urbanization and heightened global travel since World War II, the number of both dengue cases and countries reporting infection has climbed precipitously. Source: WHO; http://www.who.int/csr/disease/dengue/impact/en/index.html

**Figure:**
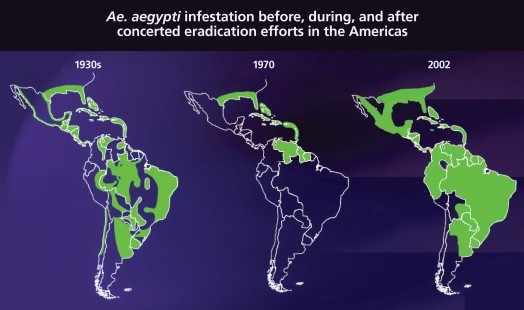
A systematic eradication program largely eliminated *Ae. aegypti* in the Americas by the 1970s. But once the program was discontinued, the vector came back stronger than ever. Source: Arias JR. 2002. Dengue: how are we doing? Washington, DC: Pan American Health Organization.

**Figure:**
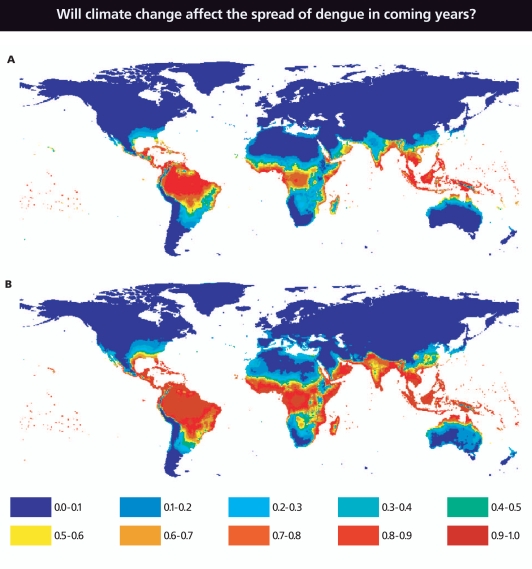
Scientists recently modeled the estimated baseline population at risk for dengue infection in 1990 (A) and in 2085 (B) using climate data for 1961–1990 and projections for humidity change—a function of climate change—for 2080–2100. Ranges above indicate percentage of the population at risk: 0–10%, 10–20%, etc. However, many scientists do not agree that climate change will appreciably alter the risk of dengue. Source: Hales S, et al. 2002. Potential effect of population and climate changes on global distribution of dengue fever: an empirical model. Lancet 360:830–834.

**Figure:**
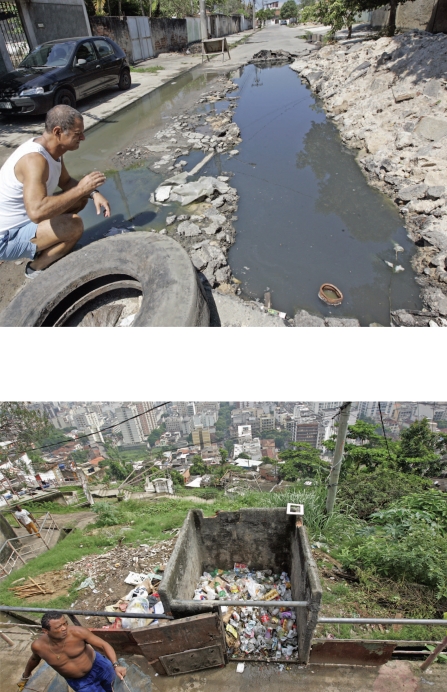
Dengue is transmitted by mosquitoes that have become perfectly adapted to the urban environment. Areas where there is poor sanitation and overcrowding (such as Rio de Janeiro, Brazil, above and below) are ripe for epidemics. According to the Brazilian Ministry of Health, Rio was the site of about half the dengue cases in an epidemic that swept this country in spring 2008.

**Figure:**
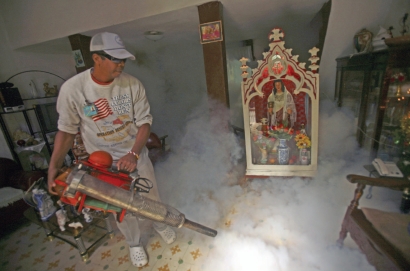
A worker fumigates a house in Old Havana, Cuba, January 2008. Control of mosquitoes with pesticides is one of the few methods currently available to rein in dengue. Systematic habitat destruction also has proved effective in the past.

